# Evidence in Coroners' Courts

**Published:** 1904-09-17

**Authors:** 


### Evidence in Coroners' Courts.

The office of coroner enjoys a traditional reputation which stretches far back in the history of the English nation. The duties attached to it are less numerous than was formerly the case, and indeed for all practical purposes are confined to the holding of inquests on the bodies of persons who have died in circumstances which render certification of the death in the usual manner impossible. This, in the enormous majority of cases, is a very simple matter, and when properly discharged merits the tribute due to useful, if not particularly dignified or difficult, public service. Unfortunately, every now and then the coroner's chair falls into the possession of one who entirely misconceives his duty and position, and instead of confining himself to his modest duties arrogates to himself a function and licence which are entirely beyond his warrant. The consequences are often inconvenient to members of the public, but they are still more prejudicial to the office which the person in question occupies. For purposes of inquiry it is, of course, essential that the coroner should have the power to summon and, if necessary, to compel the attendance of witnesses. But this right, like every other right, must be exercised with discretion. Strictly speaking, it may be assumed, a coroner may demand the presence of any citizen as a witness at any inquest he has to conduct. It is obvious, however, that there is a moral claim that he shall summon only those who are in a position to afford necessary information. In so far as he goes outside this condition he must either be a bungler in his methods or a person who uses his public office for the purpose of causing private annoyance.

We have before us newspaper reports of some recent inquests conducted at Keighley. From these we gather that the Skipton coroner, in the case of persons dying in hospital, is in the habit of summoning as a witness the nurse who was present at the time that death occurred. That may, in certain circumstances, be a proper and necessary thing to do, but these circumstances must be quite exceptional, and in no event can it be justifiable, as related in these reports, to endeavour to obtain from a nurse an opinion as to the cause of death. From what appears in the public prints this seems to be a common practice at Keighley, whilst at the same time the hospital doctor is not called as a witness. One does not, perhaps, look to the coroner's court for an exhibition of the conditions essential to judicial evidence; but to urge a nurse, who, quite rightly, declined to give an opinion as to the cause of death, to state what was written on the card placed at the head of the bed is really perilously near the ludicrous. It is, of course, an attempt to obtain by a side wind what can only be proved by the direct evidence of a medical practitioner who has either attended the person during life or has, under an order from the coroner, conducted a post-mortem examination. Behind all this one cannot but fear that there exists some friction of the responsibility for which we know nothing; though from the fact that the authorities of the Keighley Hospital have recently instructed the coroner that in future the hospital doctors are willing to attend to give evidence at inquests on in-patients without any fee in preference to the nurses being called upon, we may presume the question of payment of medical witnesses is at the foundation of the Skipton coroner's novel procedure. But we do submit that, whatever be the nature of the disagreement, its existence ought not in any way to interfere with the thorough and proper conduct of the investigations necessary in an inquiry into the cause of death. There is competent and direct testimony available in the shape of medical evidence, and the public interest demands that no attempt be made to avoid this and to substitute for it the "opinion" of unskilled persons or second-hand or hearsay reports. It would, of course, be quite wrong to say that a nurse may not at times be a necessary witness at a coroner's inquest; but her evidence can only deal with matters of fact. She is not competent to give expert testimony regarding the cause of death, and it is most unfair and improper to endeavour to compel her to do so. It is matter for surprise that an officer charged with the duty of public inquiry should sanction, and apparently should arrange, a plan of procedure in which an attempt is made to replace the competent evidence that is at hand by evidence of an inferior order. No jury should be asked to accept opinions except from those who can speak with authority, and it is far from seemly that a process having legal form should urge witnesses to assume functions which it is beyond their competence to discharge.

